# Mechanical and Physical Properties of Polyester Polymer Concrete Using Recycled Aggregates from Concrete Sleepers

**DOI:** 10.1155/2014/526346

**Published:** 2014-08-27

**Authors:** Francisco Carrión, Laura Montalbán, Julia I. Real, Teresa Real

**Affiliations:** Department of Transportation Engineering and Infrastructures, School of Civil Engineering, Polytechnic University of Valencia, 14 Camino de Vera, 46022 Valencia, Spain

## Abstract

Currently, reuse of solid waste from disused infrastructures is an important environmental issue to study. In this research, polymer concrete was developed by mixing orthophthalic unsaturated polyester resin, artificial microfillers (calcium carbonate), and waste aggregates (basalt and limestone) coming from the recycling process of concrete sleepers. The variation of the mechanical and physical properties of the polymer concrete (compressive strength, flexural strength, modulus of elasticity, density, and water absorption) was analyzed based on the modification of different variables: nature of the recycled aggregates, resin contents (11 wt%, 12 wt%, and 13 wt%), and particle-size distributions of microfillers used. The results show the influence of these variables on mechanical performance of polymer concrete. Compressive and flexural strength of recycled polymer concrete were improved by increasing amount of polyester resin and by optimizing the particle-size distribution of the microfillers. Besides, the results show the feasibility of developing a polymer concrete with excellent mechanical behavior.

## 1. Introduction

Polymer concrete (PC) is a composite material which is composed of polymeric resins that act as binder materials of aggregates and microfillers. After the addition of different additives (catalysts and accelerators), the binders undergo polymerization resulting in a hardened composite.

The primary difference, compared with cement-based concrete, apart from not containing hydrated cement, is that PC is stronger, more durable, and with lower maintenance requirements [1, 2]. However, portland cement can be used as microfiller or aggregate [3] in PC. Besides these advantages, this composite, which can reach mechanical strengths 4-5 times higher than cement-based concrete [4] keeping the modulus of elasticity in similar values [5], has good chemical resistance and water impermeability [6, 7]. For these reasons, PC is widely used in different applications of civil engineering [1, 8]. It has been used as a major component for the construction of box culverts, underground pipes, trench lines, industrial floors, also as bridge deck overlays, and in reparation tasks of damaged cement-based concrete structures.

In spite of these advantages, PC presents disadvantages that have limited its worldwide utilization. These PC disadvantages may be: expensive cost of resins used as binder agents, suitable precautions that should be applied to achieve a proper curing of PC, and need to use the high quality aggregates to produce PC, when it is compared with cement-based concrete. In order to reduce the high costs of competitive PC, together with recent environmental concern about wastes that end up in landfills, several researches [9, 10] have been conducted to analyze the properties of PC made with industrial byproducts and cement-based concrete residues acting as aggregates.

Commercial epoxy resins and commercial unsaturated polyester resins, whose good results are widely known [11], can be found among the classic resins used as binder agents. Recently, plastic wastes and bottles from polyethylene terephthalate (PET) have been used for unsaturated polyester resins production, which were used as the binder agent to produce recycled PC [6, 9, 12]. The results of these investigations were very promising. They opened an important way to reduce costs and environmental pollution caused by wastes disposal.

For their part, in order to achieve high mechanical performances by using expensive resins, high quality aggregates are commonly used. This optimum combination results in PC that fulfills such requirements [13, 14]. In this respect, a wide variety of materials are used, including quartz, silicates, gravel, limestone, calcareous, granite, clay, natural basalt, and calcium carbonate. Somewhat similar to the resin replacement, aggregates and microfillers have also been replaced by solid wastes from various industrial fields. Demolition materials from concrete and masonry wastes [15], residual glass from blasting operations [16], industry development and electrical production wastes such as fly ash or silica fume [17], crushed polymer concrete and mortar [18], rapid-cooled slag from the steel production process [10], or residual sands from foundry industries [19, 20] have been studied as mineral aggregates and microfillers in the production of recycled polymer concrete (RPC). These investigations concluded that it is feasible to produce high quality RPC based on recycled solid wastes and performances of resultant materials could be improved by optimizing different variables of the mixtures. Such variables were, in any case, aimed towards varying the ratios of resins : microfillers : aggregates used, the particle-size distributions of aggregates used, as well as their nature, and type of resins added to the mixtures.

Focusing the interest in the case of recycled aggregates from concrete and PC, the obtained results were very encouraging. Generally, as the amount of binder increases, the void ratio in PC mixtures decreases, while compressive strength and flexural strength increase. Although this is affected not only by such variables but also by grading and mixture of microfillers and aggregates used [9, 15]. Besides these facts, as a gradual increase in the content of the recycled aggregate takes place, a reduction in the modulus of elasticity of the PC can also be seen [15].

On the other hand, the use of aggregates from recycled PC has shown the feasibility of using them as a competitive alternative, compared with natural aggregates, producing only changes in mechanical strengths within 1% of significance statistic level [18]. In the case of portland cement-based concrete there is a wide range of previous investigations related with the partial or complete substitution of coarse and fine natural aggregates by recycled aggregates from concrete [21–23]. Results of those studies generally concluded that the cement-based developed concretes with recycled aggregates offered less mechanical strengths and lower elastic modulus.

Recently, the growing environmental awareness on the reuse of solid wastes from disused infrastructures, alongside the difficulties to obtain high-quality natural aggregates, leads to the study and incorporation of these waste materials as PC components. In this research, the fundamental sources of aggregates are the replaced concrete sleepers or those whose manufacture has been faulty.

Railway sleepers are essential elements in railroads; their main role is to distribute loads from the railway vehicles to the underlying ballast bed. Particularly, in the case of high-speed railroads, loads are very demanding. Therefore, materials that compose these elements should provide the maximum performance [24]. In the case of concrete sleepers, the most used composition for their manufacture in Spain is siliceous aggregates, limestone, and basalt. Their mechanical properties are very suitable; consequently, they may provide an excellent source of high-quality recycled aggregates.

From these considerations, this paper presents the results of experimental research on PC made with unsaturated polyester resin, microfillers, and recycled aggregates from crushing, cleaning, screening, and sieving replaced concrete sleepers or faulty ones. Furthermore, it also attempts to assess the mechanical properties of new PC, by varying the resin content, the nature of the recycled aggregate, and the particle-size distribution of microfillers blended.

## 2. Materials and Methods

### 2.1. Materials Used

#### 2.1.1. Resin

Unsaturated orthophthalic polyester resin available as a commercial product HEGARDT H-76.1 was used to manufacture all mixtures developed in the present research.  Table 1 shows the main physical properties of the used binder, according to the manufacturer. This polyester resin offers a low exothermic peak (200°C) and low volume shrinkage, therefore it is especially recommended for the production of PC. Methyl ethyl ketone peroxide (MEKP) in dimethyl phthalate (DMP) was used as initiator for this selected polyester resin.

#### 2.1.2. Aggregates

Three different fine-size aggregates with a nominal maximum size of 4 mm, according to European Standard EN 12620:2009, were used: natural basalt (NBA), obtained from crushed basaltic rocks; recycled basalt (RBA), obtained from replaced and defective concrete sleepers; and recycled limestone (RLA) obtained following the same procedure that for RBA.

**Figure 1:**
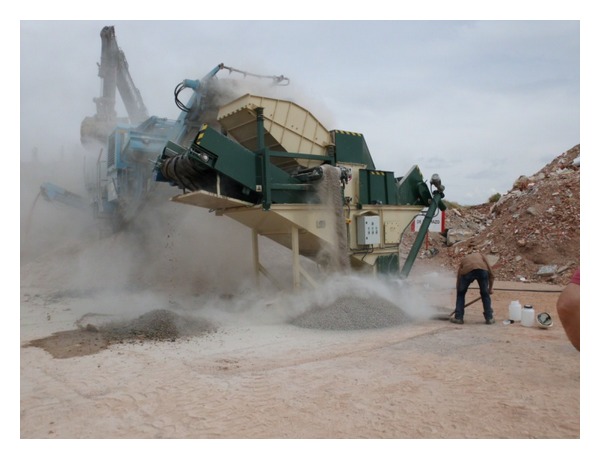
Recycling railway sleepers process.

The process used to recycle railway sleepers consisted of crushing the whole element, as indicated afterwards. Firstly, the sleeper was crushed. Secondly, the steel and iron particles were removed using a magnetic process. Thirdly, the resultant granular materials were screened to obtain well-graded aggregates. When this process was completed, the treated material was crushed again so as to produce the desired particle-size distribution, and air flow currents were applied to remove dusty particles. Finally, the resultant aggregates were cleaned with water and sieved. In spite of this complex treatment, mortar appeared attached to the particles at the end of the process. Absorption tests and frost/thaw cycle resistant tests were carried out to quantify the remaining mortar attached to the aggregates.  Figure 1 shows a photograph of the process described.

It should be noted that water absorption of RBA (8.7%) was higher than RLA (6%); this fact indicated that RBA had more attached mortar (from high resistant portland cement concrete) because of its rougher surface. In the frost/thaw cycle resistant tests (EN 14617-5:2012), RBA aggregate reduced its weight by 0.9% and RLA by 0.3%. These results were in accordance with those obtained in the absorption tests. For its part, density values were similar for all of the recycled aggregates (*ρ*_RBA_ = 2.58 g/cm^3^ and *ρ*_RLA_ = 2.48 g/cm^3^).

**Figure 2:**
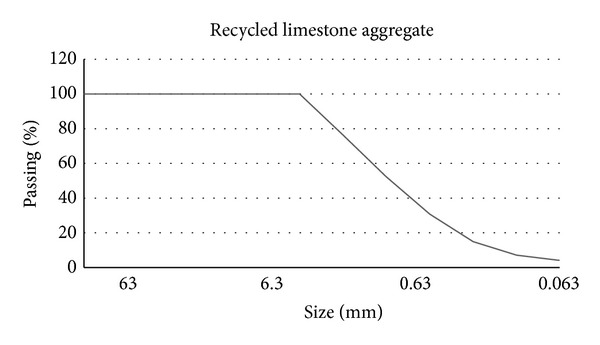
Limestone recycled aggregate gradation.

**Figure 3:**
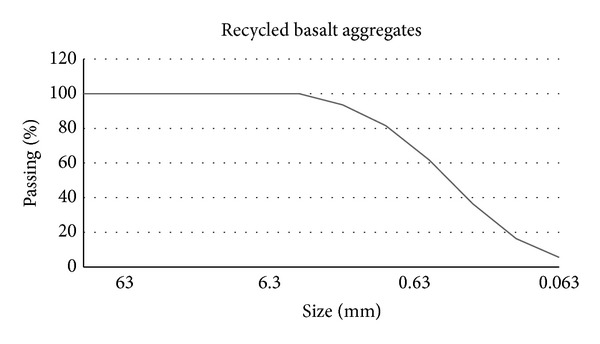
Basalt recycled aggregates gradation.

**Figure 4:**
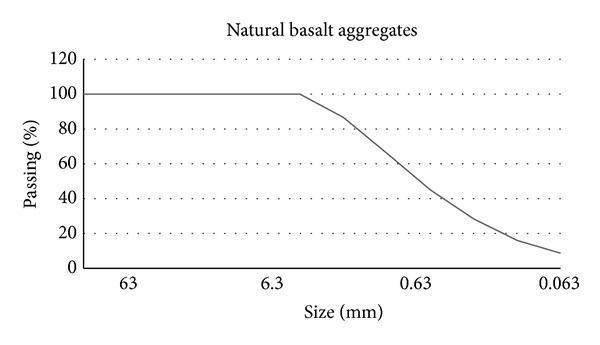
Natural basalt aggregate gradation.

Different types of aggregates were used to analyze their influence in the PC mechanical performance depending on the origin and nature of them. Particle-size distributions of these aggregates were not considered since they are practically equal in size, as it can be seen in Figures 2, 3, and 4 in which aggregates gradation appears.

#### 2.1.3. Microfillers

In order to enhance the bonding strength between resins and inorganic aggregates, three different microfillers from calcium carbonate (CaCO_3_) were used [25]. Individual microfillers had the same origin, only the particle-size distributions were modified and, therefore, their bulk densities and moisture contents. Physical properties of the three microfillers: MF1 (maximum grain size of 500 *μ*m), MF2 (maximum grain size of 100 *μ*m), and MF3 (maximum grain size of 1 mm), are given in Table 2.

**Figure 5:**
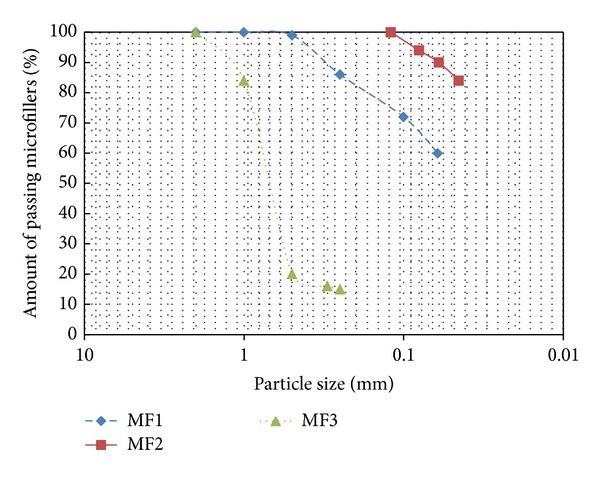
Particle-size distribution of the individual microfillers used in the study (MF1, MF2, and MF3).

A comparison of the three different particle-size distributions is shown in Figure 5. This study blends the three individual microfillers for the production of PC with variations in the finest particles (<0.045 mm). Thus, by mixing of various types of microfillers, intended to increase the finest particles contents in the mixtures, in order to obtain a lower void content and keep constant the rest of parameters, up to two different microfillers blends were produced. This allowed the study of this effect on mechanical properties of the PC [26].

### 2.2. Methods of Experiment

Six mixtures of PC were developed with different origin of aggregates (natural basalt, recycled basalt, and recycled limestone), different particle-size distribution for calcium carbonate microfillers blends, and three different contents of unsaturated polyester resin (11%, 12%, and 13% of the total weight of the mixture). Those typical values were adopted from literature review [7, 9, 11] and adapted to the present study.  Table 3 shows the six different mixtures combinations of PC developed for this research.

As it can be seen in Table 4, different laboratory tests were needed in order to obtain the mechanical and physical properties of each mixture. Polymeric concrete specimens of each mixture were tested for the following [27–29].

- Compressive strength which is measured based on EN 14617-15:2006.
- Flexural strength which is determined based on EN 14617-2:2008.
- Static modulus of elasticity which is determined based on EN 14617-15:2006.
- Density and water absorption which are measured based on EN 14617-1:2013.

**Figure 6:**
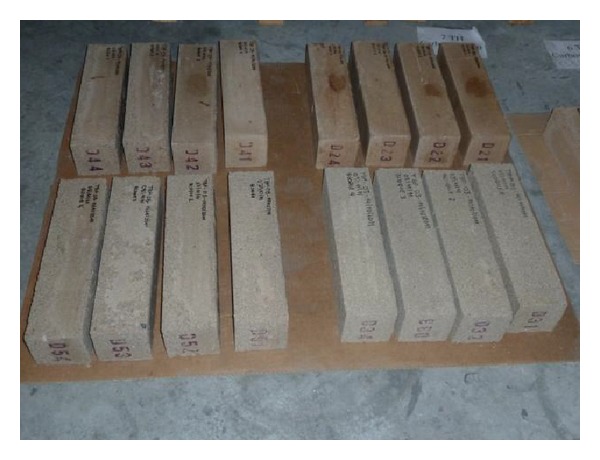
Specimens used for the flexural strength.

**Figure 7:**
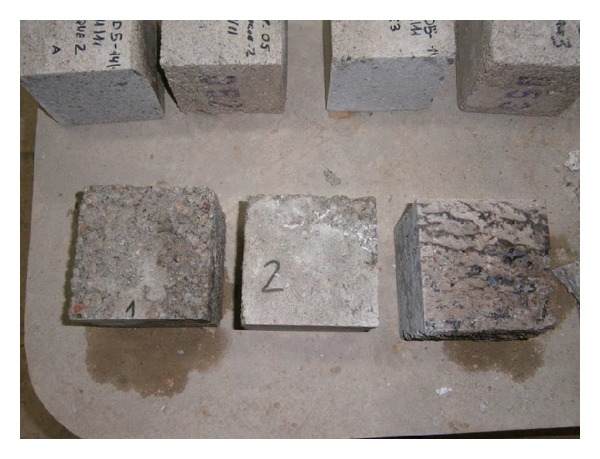
Specimens used for the compression strength.

Figures 6 and 7 show the specimens used for the tests.

These six dosages were designed with the aim of analyzing four case studies by comparisons between them. In the first case, the variable of study was the nature of the recycled aggregate from concrete sleepers (limestone or basalt), while keeping the minimum workability resin content (11 wt%) and a particle-size distribution of microfiller with a low content of fine particles (a blend of MF1 and MF3). Thus, this resulted in a comparison between RLA-11 and RBA-11 mixtures.

The second group of study involved the analysis of the mechanical performance of PC made with recycled basaltic aggregates (from replaced and faulty concrete sleepers), with the variations of the resin amount (from 11 wt% to 13 wt%), but keeping constant the amount of recycled aggregate and particle-size distributions of microfillers. Thus, this case led to a comparison between RBA-11MF, RBA-12MF, and RBA-13MF.

Another case was based on the analysis of the mechanical behavior of the PC from the modification of particle-size distribution of the two blends of microfillers. A comparison between RBA-11 and RBA-11MF dosages was obtained from the increase of the content of the fine particles smaller than 0.045 mm (approximately twice), but maintaining the resin contents (11 wt%) and the nature of recycled aggregate (RBA).

The study involved only the performance of PC with the addition of natural basaltic aggregate (21.75 wt%) to complement the use of recycled basaltic aggregate (21.75 wt%). This was done while keeping constant the amount of resins (13 wt%) and the particle-size distribution of microfillers (a blend of MF1, MF2, and MF3). Thus, this led to a comparison between RBA-13MF and NBA-13MF.

Unsaturated polyester resin, catalysts, and accelerating agent were weighed and blended in a conventional rotator mixer. Microfillers and aggregates, previously weighed in an appropriate proportion, were carefully added to the mix. Once the mixture was uniformly developed, specimens were manufactured.

In the case of the compressive strength, modulus of elasticity, density, and water absorption, cubic specimens measuring 10 × 10 × 10 cm were used. Six specimens were cast for each composition to conduct these tests, except for modulus of elasticity determination (four specimens). Cubic molds were filled with polymer concrete, and they were compacted and vibrated to obtain homogeneous specimens. The equipment used for this process was a vibrator table during 80 seconds (EN 12390-2:2009). All this process was done under ambient temperature (20°C). After the manufacture of the specimens, they were cured in an oven under 70°C for 24 hours, until they offered a constant weight. Then, the specimens were kept under 20°C and ambient moisture for 28 days. Samples were tested at 28 days since they were produced. On the other hand, for the flexural strength test, six prismatic specimens measuring 10 × 10 × 40 cm were cast for each composition.

The specimens produced to obtain the flexural strength were prepared following a similar process. The only difference was the temperature of the curing process: specimens were introduced into an oven at 40°C until they offered a constant weight. Samples were tested at 28 days of age under 20°C temperature. Results given in this paper represent the mean of the individual values for each PC specimen.

## 3. Results and Discussion

After performing the tests for prepared PC specimens, experimental results were obtained for the flexural strength (*f*_fl_), compressive strength (*f*_*c*_), modulus of elasticity (*E*), density (*ρ*), and water absorption (Abs). Table 4 shows the average values for the results obtained in the experimental tests for each mixture.

### 3.1. Nature of Recycled Aggregates

**Figure 8:**
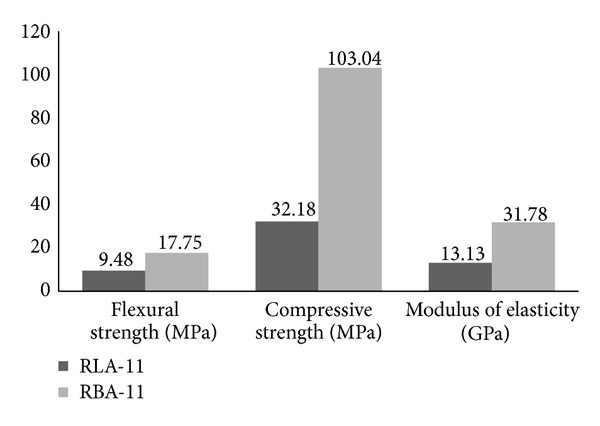
Comparison of the obtained mechanical properties from RLA-11 and RBA-11.

Firstly, recycled limestone aggregates (RLA) or recycled basaltic aggregates (RBA) have been studied focusing on the benefits they gave to the mechanical properties of the final PC.  Figure 8 shows the mechanical properties comparison and, then, physical properties are shown in Table 5.

On average, the difference could be quantified by flexural resistances 53.41% higher with the use of RBA over the use of RLA. These results indicated an improvement of performances in bending when basalt recycled aggregates from concrete sleepers were used.

The compressive strengths obtained in standard tests were much higher in the case of PC prepared with RBA (103.04 Mpa). Compressive strength was 68.77% lower in the PC prepared with RLA, compared to those made with RBA.

Since density of RLA (2.48 g/cm^3^) was slightly lower than density of RBA (2.58 g/cm^3^), densities resultant from the manufactured PC (RLA-11 and RBA-11) reflected this fact. Even so, it should be noted that density obtained was similar in both cases (approximately difference of 5%).

Furthermore, the extreme difference between the modulus of elasticity resultant in both manufactured PCs, confirmed that the use of recycled basaltic aggregate provided a better mechanical performance to PC.

Consequently, it could be seen that the use of recycled aggregate from basaltic nature offered better mechanical performance compared to the recycled limestone aggregate. Even though the RBA had higher water absorption value that could weaken the bond with the resins [18]. Furthermore, this fact suggested that the better mechanical performance of basaltic aggregate led PC to achieve similar mechanical properties to those made with high-quality natural aggregates [4, 9, 18, 26, 30] (*f*_*c*_ ≈ 90 MPa, *f*_fl_ ≈ 25 MPa and *E* ≈ 30.000 MPa).

More resistant basaltic origin of RBA compared to the recycled limestone aggregate (RLA) contributed to improve the mechanical performance of the polymer concrete.

Since it was estimated that RBA particles had more quantity of mortar attached to their surfaces due to their rougher surface (compared with RLA particles), this suggests that this fact could play a key role in the mechanical characteristics of the produced polymer concretes.

According to previous literature [8], resistant microstructure of polymer concrete is based in a thin layer of matrix, consisting in a mix of resin and microfillers, which coat the coarse aggregates particles. The rougher and irregular surface could improve the adhesion between the matrix and the coarse aggregate, which resulted in improved compressive and flexural strengths.

More attached mortar is beneficial for adhering the matrix to the aggregates and, thus, for improving the distribution of tensions. However, the quantity of the attached mortar will be limited, and it must be studied in future researches in order to better understand this performance. Nevertheless, it is necessary to consider that the existence of attached mortar could result in durability problems. In the case that the aggregates used to produce PC were extremely reactive with the alkali of cement, the produced PC could be damaged by the expansive reactions [31]. Consequently, precautions should be taken into account in the choice of the aggregates, when recycled concrete is used to produce PC. On the other hand, expansive alkali-aggregate reactions which the recycled concrete aggregates have the potential for may not be an important risk in this situation due to the fact that these reactions occur under availability of moisture, while polymer concretes usually have very low water absorption or permeability.

### 3.2. Resins Content Variation

**Figure 9:**
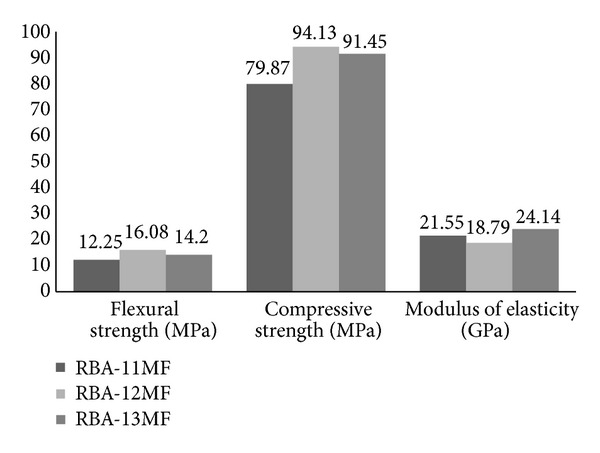
Comparison of the obtained mechanical properties from RBA-11MF, RBA-12MF, and RBA-13MF.

According to the results shown above, the best performance of PC made with recycled aggregates from the treatment of replaced or faulty concrete sleepers was obtained by adding RBA in the mixtures. This was the reason why RBA was used to investigate the effects on mechanical properties of PC with the variation of resin content.  Figure 9 shows flexural strength, compressive strength, and modulus of elasticity from RBA-11MF, RBA-12MF, and RBA-13MF specimens. These concretes were produced by keeping the same microfillers ratios and aggregates, only modifying the total resin contents from 11 wt% to 13 wt%.

Table 6 exposes the results obtained for the density and water absorption from RBA-11MF, RBA-12MF, and RBA-13MF specimens

As illustrated by the results, the general trend observed with the increase of the amount of resin was an improvement of the mechanical performance of PC, to certain extent. The highest flexural strength resulted from PC with 12 wt% resin (16.08 MPa). The higher the added quantity of resin was, the higher the resultant flexural strength was. But when this content reached 13 wt% (RBA-13MF), the flexural strength did not increase markedly, and contrary to what was expected, it decreased (14.2 MPa). Consequently, a loss of effectiveness with the highest amount of added resin (13 wt%) appeared.

A similar trend occurred with the compressive strength, as it can be seen in Figure 9. The highest value (RBA-12MF) was higher than those resulting from RBA-11MF (79.87 MPa) and RBA-13MF (91.45 MPa). Consequently, this trend indicated that, from the standpoint of mechanical performance, with the nature and amount of filler employed, the better resin content was 12 wt%.

Since the specific gravity of polyester resin was lower than that of the aggregates and microfillers, along with the increase of the quantity of resin, a slight decrease was noticed in densities of PC [4, 16, 30].

PC water absorption was decreased along with the increase of the amount of resin. However, when the content exceeded 12 wt% (RBA-12MF), this effect was less effective and water absorption experienced a slight increase. RBA-13MF resulted in a 0.19% of water absorption, compared with 0.11% of RBA-12MF. Water absorption evolution can be explained taking into account that 11 wt% resin content is not enough and aggregates appear in the mixture surface. This situation does not occur with 12 wt% and, thus, the water absorption of this mixture is significantly reduced. In this way, resin contents greater than 12 wt% cause a slight increase in the water absorption value. This is explained by the major resin content since the resin used presents water absorption, as shown in Table 1.

According to previous researches [5, 30], an increase in resin content causes gradual stiffening of the mechanical properties of PC (increases the modulus of elasticity), to some extent (optimum resin content), from which there is a loss of stiffness. This was exactly noticed in the present research, but when the optimum content (RBA-12MF) was reached from the standpoint of strength, the modulus of elasticity was lower than that resulted from RBA-11MF and RBA-13MF.

The results obtained from conducted tests suggested that, according to the content of microfillers used, the specific surface that they provide for good adhesion between resin and aggregates was achieved with 12 wt% of resin. Adding greater amounts of resin (13 wt%), did not produce a proper filling of voids and the mixture was not homogeneous, causing weakness.

These results were in line with those obtained in previous researches that made use of recycled concrete to produce PC [15]. The deficient filling of voids in the PC matrix could be attributed to the mortar attached to the particles of the recycled aggregate. This mortar had a more porous structure, compared with the original of the RBA or RLA, consequently, the use of more amounts of resin results in a loss of efficiency. This phenomenon is due to the filling of the voids in the attached mortar to the detriment of the formation of the thin matrix layer around the aggregates.

Thus, additionally to the durability problem mentioned above, the mortar attached to the recycled aggregates could modify the optimum amount of resin in the production of PC, in comparison with PC produced with natural aggregates. This effect could lead to a loss of effectiveness in the use of higher amounts of resin.

### 3.3. Variation of the Particle-Size Distribution of Microfillers

**Figure 10:**
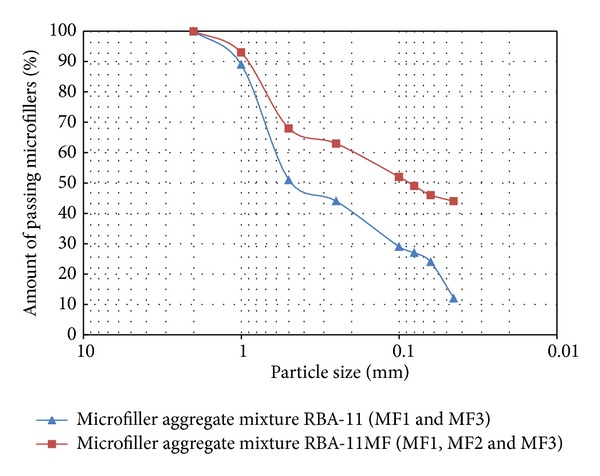
Particle-size distribution of the different microfillers blends used in the PC tested (RBA-11MF and RBA-11).

In order to study how the particle-size distributions and grain sizes of microfillers affected the mechanical performance of PC, two dosages were tested: RBA-11 and RBA-11MF. Both mixtures had the same resin content (11 wt%), the same type of recycled aggregate (RBA), and the same ratio (44.5 wt%). Only the size-distributions of the finest particles were modified. Particle-size distributions of the microfillers blends used in the tests are given in Figure 10.

**Figure 11:**
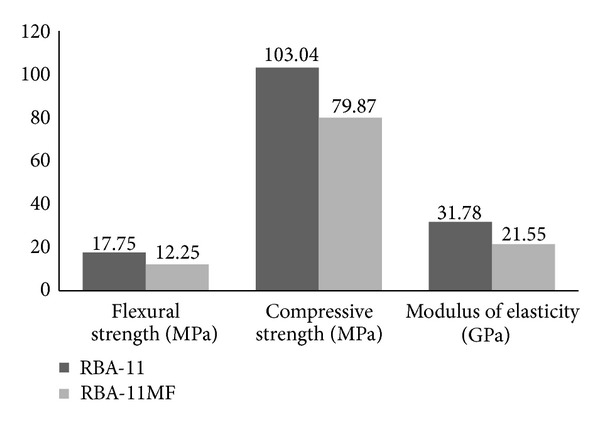
Comparison of the obtained mechanical properties from RBA-11 and RBA-11MF.

As it can be seen from Figure 10, the amount of particles smaller than 1 mm was higher in RBA-11MF. Particles which were smaller than 0.045 mm were doubled in quantity.  Figure 11 and Table 7 show the compared tests results from RBA-11 and RBA-11MF.

The higher the fine particle contents in the microfillers, the lower the flexural strength in PC. On average, flexural strength from test results of RBA-11 was 30.9% higher than flexural strength from RBA-11MF. A similar effect was also observed for compressive strength; in such situation the reduction from RBA-11 to RBA-11MF was approximately 22.5%. Therefore, it may be noted that particle-size distributions of microfillers and the amount of the fine particles in such materials influenced notably the mechanical strength of PC [32]. These results have been different to those obtained in some previous investigations [15]. On such studies it was concluded that higher amounts of finer particles provided a thicker matrix around the coarse aggregates and, consequently, an improved strength in the PC. Contrary to this, in the present research the use of higher amounts of finer particles has led to lower performances of PC. This effect could be attributed to a poor gradation of the microfillers blend used in RBA-11MF, compared with the microfillers blend used in RBA-11.

The poor microfiller gradation causes increase of the void ratio, thus the PC mixture is not homogeneous and it causes worsening of the mechanical properties of PC. This effect should be investigated deeply in order to demonstrate this reasoning, when recycled aggregates are used in the production of PC.

The conducted tests of density from both mixtures (RBA-11 and RBA-11MF) gave similar values (2.20 g/cm^3^ and 2.22 g/cm^3^, resp.). Aside from this, observing the elastic behavior of both polymer concretes under testing, the lower the particle size in microfillers (RBA-11), the stiffer the PC resultant, keeping constant the total microfillers ratio in the mixture (44.5 wt%) and the resin content (11 wt%).

In addition to these results, the effectiveness of improving the mechanical performance of PC by changing the particle-size distribution of microfillers compared with the increase in the amount of resin was studied. To carry out this additional investigation, a comparison between RBA-11, RBA-11MF, RBA-12MF, and RBA-13MF was done. From the results given in Table 7, it should be noted, in terms of mechanical strength, that it was more cost-effective to add well graded microfillers mixtures (smaller amount of finer particles) and lower resin amounts than to make use of poor graded microfillers mixtures (larger amount of finer particles) and richer resin contents. These results were more representative in the specific case of comparing a PC with lower resin content and well-graded microfillers mixture (RBA-11) with a PC composed by a higher content of resin and a poor-graded microfillers mixture (RBA-13MF). Results from this comparison showed flexural strengths 25% higher and compressive strengths 12.7% higher.

### 3.4. Effect of Adding Natural Aggregate as Supplement

In order to develop a higher performance PC, made with recycled aggregates from replaced concrete sleepers, a last variable was analyzed. The use of complementary natural basaltic aggregate (NBA) from crushed basalt rock was studied.

**Figure 12:**
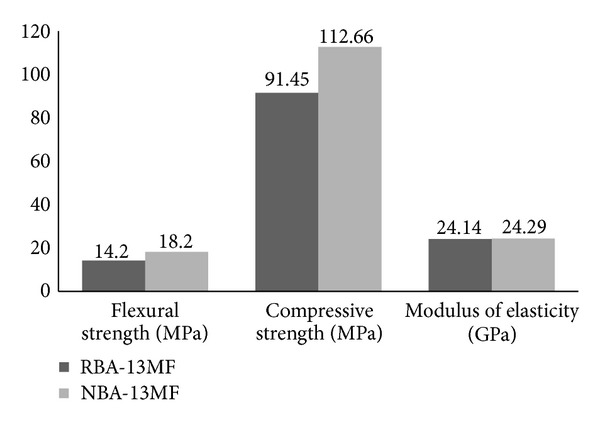
Comparison of the obtained mechanical properties form RBA-13MF and NBA-13MF.

This aggregate was added in combination with the RBA, in a replacement of 50%, which resulted in a natural aggregate total ratio of 21.75 wt%. Thus, the PC developed by this method was NBA-13MF which had the same conditions as RBA-13MF, except for the use of natural basaltic aggregate. The main tests results from both evaluated PCs are given in Figure 12 and Table 8.

Modulus of elasticity (*E*_NBA-13MF_ = 24292.56 MPa and *E*_RBA-13MF_ = 24141.98 MPa) and water absorption (Wabs_NBA-13MF_ = 0.20% and Wabs_RBA-13MF_ = 0.19%) remained constant. The reason for this is that natural aggregate which presents different aggregate gradation was used and, thus the mixture conditions were changed. The flexural strength was improved by replacing part of recycled aggregate with natural aggregate. In particular, this improvement could be quantified by 28.2%. Relative to the compressive strength, such improvement was quantified by 23.2%. Since the specific weight of natural aggregates was higher than recycled aggregates, the resultant density of NBA-13MF was slightly higher than the density of RBA-13MF. These results were similar to those reported by previous investigations [9] where recycled concrete aggregates were used.

It is worth to mention that in this study, three mechanical properties (compressive strength, flexural strength, and modulus of elasticity) and two physical properties (density and water absorption) of PC were studied, made with the use of recycled aggregate from portland cement concrete.

In order to extend and standardize the use of recycled concrete in the production of high strength PC, it would be adequate to analyze some additional variables. Particularly the behavior of the produced PC when it is exposed to aggressive chemical environments and high temperature expositions, given the use of this material in chemical applications (buried pipes, industrial floors, etc.).

Moreover, the use of PC in the manufacture of sewer pipelines for trenchless construction is well known. For this reason, a study of permeability and acid attack would be desirable in future researches.

The high strength achieved in the PC developed in this study makes it capable to be used in the construction of any structural element: beams, slabs, pipes, or any precast element of PC. In this case, the interest of study should be focused on the shrinkage experimented by the material in order to avoid potential damages. This is in line with the precautions that must be taken into account when recycled aggregates are used in the production on PC because of alkali reactions of the attached mortar with the aggregates used.

## 4. Conclusions

This study identified the possibility of producing high-performance polymer polyester concrete by using recycled aggregates from crushed, cleaned, screened and sieved, replaced, or faulty manufactured concrete sleepers. Besides this, the physical and mechanical properties of the recycled polyester polymer concrete produced were analyzed under the variation of origin of the aggregates, particle size-distribution of microfillers blends, and amount of binder used. The following conclusions may be drawn from the results and comparisons of this research.

- It was determined that mechanical properties of PC were improved by using recycled basaltic aggregate instead of adding recycled limestone aggregate. A better mechanical behavior of RBA aggregate was reported, even though it had higher water absorption, compared with RLA aggregate.
- The effect of adding higher amounts of unsaturated polyester resin, to the PC made with recycled basaltic aggregates, caused a general increase of the mechanical strengths and stiffening. Additionally a gradual increase of resin (>12%) content caused less absorbent and lighter PC.
- Granulometric curve of microfillers played a key role in the improvement of mechanical performances of the recycled PC. An excessive amount of the finest particles (<0.045 mm) prevented an adequate penetration of the binder into the mineral structure of the mixture. Besides this, it was reported that by optimizing the grading curve of microfillers, a better performance could be achieved if the results were compared with the results obtained by varying the content of resins.
- If improved mechanical performance is required, part of the recycled basaltic aggregate could be replaced by natural basaltic aggregate (25 wt% in this study), resulting in an improvement of the compressive strength, over 23.2% higher.
- The main aim of this study was to achieve the highest compressive and flexural strength and to observe how these parameters changed with the variation of some factors. However, a more economical approach could be achieved if less resin, improved microfillers choice, and recycled aggregate were used. Consequently, with these results the required strength may meet with a cost effective PC.

## Figures and Tables

**Table 1:** Physical properties of binder used.

Density (g/cm^3^, 25°C)	Viscosity (Pa*·*s, 25°C)	Styrene content (%, 25°C)	Water absorption (%)
1.10	0.2	34	0.2

**Table 2:** Physical properties and chemical composition of the three microfillers used.

Type	Max. grain size (mm)	Bulk density (g/cm^3^)	Moisture (%)
MF1 (CaCO_3_)	0.5	0.93	0.06
MF2 (CaCO_3_)	0.1	0.86	0.06
MF3 (CaCO_3_)	1	1.35	0.2

**Table 3:** Mix proportions of PC containing recycled aggregates from concrete sleepers.

Mixture	Polyester resin (wt%)	Aggregates (0/4 mm)			Microfillers (<1 mm)		
		NBA (wt%)	RBA (wt%)	RLA (wt%)	MF1 (wt%)	MF2 (wt%)	MF3 (wt%)
RLA-11	11.0	0.0	0.0	44.5	17.8	0.0	26.7
RBA-11	11.0	0.0	44.5	0.0	17.8	0.0	26.7
RBA-11MF	11.0	0.0	44.5	0.0	13.35	13.35	17.8
RBA-12MF	12.0	0.0	44.0	0.0	13.2	13.2	17.6
RBA-13MF	13.0	0.0	43.5	0.0	13.05	13.05	17.4
NBA-13MF	13.0	21.75	21.75	0.0	13.05	13.05	17.4

**Table 4:** Test results: mechanical properties, density, and water absorption (and standard deviation for each mean value).

Mixture	*f*_fl_ (MPa)	*f*_*c*_ (MPa)	*E* (MPa)	*ρ* (g/cm^3^)	Water absorption (%)
RLA-11	9.48 ± 1.16	32.18 ± 2.86	13130.33 ± 2.79	2.093 ± 0.0025	—
RBA-11	17.75 ± 2.23	103.04 ± 3.7	31783.94 ± 3.12	2.199 ± 0.0062	—
RBA-11MF	12.25 ± 1	79.87 ± 1.59	21553.93 ± 3.8	2.221 ± 0.0064	0.44 ± 0.118
RBA-12MF	16.08 ± 1.15	94.13 ± 3.02	18787.06 ± 2.85	2.218 ± 0.0025	0.11 ± 0.025
RBA-13MF	14.2 ± 0.51	91.45 ± 2.08	24141.98 ± 4.32	2.212 ± 0.0072	0.19 ± 0.025
NBA-13MF	18.2 ± 1.68	112.66 ± 3.3	24292.56 ± 1.27	2.309 ± 0.0037	0.20 ± 0.000

**Table 5:** Physical properties.

Mixture	*ρ* (g/cm^3^)	Water absorption (%)
RLA-11	2.093	—
RBA-11	2.199	—

**Table 6:** Physical properties.

Mixture	*ρ* (g/cm^3^)	Water absorption (%)
RBA-11MF	2.221	0.44
RBA-12MF	2.218	0.11
RBA-13MF	2.212	0.19

**Table 7:** Physical properties.

Mixture	*ρ* (g/cm^3^)	Water absorption (%)
RBA-11	2.199	—
RBA-11MF	2.221	0.44

**Table 8:** Physical properties.

Mixture	*ρ* (g/cm^3^)	Water absorption (%)
RBA-13MF	2.212	0.19
NBA-13MF	2.309	0.2
